# Diabetes and Cancer: Metabolic Association, Therapeutic Challenges, and the Role of Natural Products

**DOI:** 10.3390/molecules26082179

**Published:** 2021-04-10

**Authors:** Wamidh H. Talib, Asma Ismail Mahmod, Sara Feras. Abuarab, Eliza Hasen, Amer A. Munaim, Shatha Khaled Haif, Amani Marwan Ayyash, Samar Khater, Intisar Hadi AL-Yasari, Lina T. Al Kury

**Affiliations:** 1 Department of Clinical Pharmacy and Therapeutic, Applied Science Private University, Amman 11931-166, Jordan; asmamahmod1212@gmail.com (A.I.M.); saraarab97@yahoo.com (S.F.A.); elyzahasan97@gmail.com (E.H.); am00er97@gmail.com (A.A.M.); shaza-haif@hotmail.com (S.K.H.); amaniayyash@gmail.com (A.M.A.); samar_ktr@asu.edu.jo (S.K.); 2 Department of Genetic Engineering, College of Biotechnology, Al-Qasim Green University, Babylon 00964, Iraq; entesar@biotech.uoqasim.edu.iq; 3 Department of Health Sciences, College of Natural and Health Sciences, Zayed University, Abu Dhabi 144534, United Arab Emirates; Lina.AlKury@zu.ac.ae

**Keywords:** hyperglycemia, cancer, Warburg effect, alternative therapies, obesity, metabolic syndrome, hyperinsulinemia, cancer metabolism, oxidative stress, natural products

## Abstract

Cancer is considered the second leading cause of death worldwide and in 2018 it was responsible for approximately 9.6 million deaths. Globally, about one in six deaths are caused by cancer. A strong correlation was found between diabetes mellitus and carcinogenesis with the most evident correlation was with type 2 diabetes mellitus (T2DM). Research has proven that elevated blood glucose levels take part in cell proliferation and cancer cell progression. However, limited studies were conducted to evaluate the efficiency of conventional therapies in diabetic cancer patients. In this review, the correlation between cancer and diabetes will be discussed and the mechanisms by which the two diseases interact with each other, as well as the therapeutics challenges in treating patients with diabetes and cancer with possible solutions to overcome these challenges. Natural products targeting both diseases were discussed with detailed mechanisms of action. This review will provide a solid base for researchers and physicians to test natural products as adjuvant alternative therapies to treat cancer in diabetic patients.

## 1. Introduction

Diabetes mellitus is a class of metabolic disorders characterized by prolonged periods of hyperglycemia. According to the World Health Organization (WHO), in the year 2014, 422 million people worldwide suffered from diabetes, and 1.6 million deaths were directly related to diabetes in the year 2016. Moreover, these statistics have been rising slowly over the last few decades [1]. Diabetes mellitus (DM) is displayed as either type 1 diabetes mellitus (result from total insulin deficiency due to beta-cell death follow an autoimmune disease) or type 2 Diabetes Mellitus (predominantly resulting from insulin resistance, rendering target cells unable to effectively respond to insulin and so unable to utilize blood glucose for energy). It is well known that diabetes mellitus increases the risk of developing a variety of severe life-threatening health complications, resulting from the disruption and impairment in the function of several organs (kidneys, hearts, skin, blood vessels, or nerves), leading to both microvascular and macrovascular complications which include nephropathy, diabetic retinopathy, and neuropathy, as well as atherosclerosis, hypertension, and stroke. These diabetic vascular complications are responsible for the majority of mortality in diabetic patients [2]. In addition to these complications, elevated blood glucose levels have been shown to stimulate cancer cell proliferation and progression [3]. Studies have shown a strong correlation between diabetes mellitus and carcinogenesis and the most evident correlation is reported with type 2 diabetes mellitus (T2DM). Nevertheless, In type 1 diabetes mellitus (T1DM), the risk of carcinogenesis has also been identified but is less evident compared to that with T2DM [4]. However, in both types of diabetes mellitus cancer incidence tends to be increased [5]. Cancer is the second leading cause of death worldwide and in the year 2018, approximately 9.6 million deaths were attributed to cancer, and nearly one in six deaths around the world are caused by cancer [6]. Both diabetes and cancer have a dramatic detrimental effect on both the mortality rate and the quality of life, and the simultaneous rise in incidence rates of both diseases has encouraged the research community to search into a possible correlation in terms of pathophysiological pathways and/or the common climate [7]. Hyperglycemia causes epigenetic alterations by several mechanisms including DNA methylation and chromatin remodeling, resulting in abnormal gene expression. Moreover, in cancer, abnormal gene expression causes tumor growth by increasing the metastases, proliferation, and chemoresistance of cancer cells [8]. The proliferation of cancer cells which is induced by hyperglycemia/diabetes occurs indirectly by mediating the following processes (1) insulin and insulin-like growth factor 1 (IGF-1), (2) secretion of leptin/adiponectin, (3) inflammatory responses, (4) production of reactive oxygen species (ROS; oxidative stress) and (5) immune abnormalities (platelet activation) [9]. In addition to a direct correlation between impaired glucose tolerance/diabetes and the initiation of cancer, proliferation and invasiveness may occur due to hyperglycemia [10]. Many epidemiological reports have shown that diabetes is positively associated with several types of cancers including breast, colorectal, endometrial, liver and pancreatic cancers. On the other hand, a decreased prevalence of prostate cancer has been observed in diabetic patients [11]. Ben et al. found a significant association between DM and pancreatic with an inverse correlation between the duration of DM and the occurrence of pancreatic cancer in both sexes, thus observing the greatest risk of developing pancreatic cancer in patients with a duration of DM shorter than 1 year after diagnosis [12]. Similar findings were observed in another study [13]. Cancer risk in diabetic patients is 20% higher based on a meta-analysis of 20 studies [14]. The analysis of data obtained by Hulda Hrund Bjornsdottir et al. on 450,000 people with type 2 diabetes and more than 2 million people without diabetes in Sweden between 1998 and 2014 was unable to show a cause-and-effect relationship. Nevertheless, people with blood sugar disease were observed to have a higher risk of developing several types of cancer including a 231% higher risk of hepatocellular cancer, a 119% higher risk of pancreatic cancer, and a 78% higher risk of uterine cancer compared to those without type 2 diabetes. Additionally, people with diabetes had an elevated risk of kidney cancer (45% higher), stomach cancer (21% higher), cancer of the gallbladder and bile duct (32% higher), and penile cancer (56% higher). Moreover, the incidence of colorectal cancer and bladder cancer was also 20% higher, while the breast cancer risk was 5% higher [15]. Besides the increased risk of cancer by hyperglycemia/diabetes, several meta-analysis studies have also linked diabetes with a higher incidence of poor post-treatment prognosis in diabetic cancer subjects [16]. In addition, the effect of hyperglycemia on breast cancer chemotherapy resistance have been shown in recent studies [17]. Although epidemiological studies have shown that cancer mortality in diabetic patients is relatively increased, it is still unclear if this is a consequence of hyperglycemia and hyperinsulinemia (growth-promoting effect on cancer cells), poor health conditions attributable to diabetes comorbidity, or a combination of all these factors [18]. There is strong evidence that cancer patients with diabetes are treated less aggressively or are expected to be less likely treated with modified anti-cancer therapy than non-diabetic cancer patients [19]. Some anti-diabetic drugs, such as metformin, have been shown to reduce the incidence of cancer in diabetes patients, enhance the efficacy of anti-cancer drugs and also has shown favorable survival in cancer patients [16]. This review presents a detailed discussion of the major metabolic changes in cancer cells that maintain their development. Furthermore, since diabetes is a metabolic syndrome we will discuss the metabolic linkage between diabetes and cancer. We also will describe therapeutic challenges facing treating diabetic patients with cancer and suggested therapies. Finally, we will explore various natural products that have the capacity to target both diseases and the mechanisms of action on both diseases.

## 2. Cancer Altered Metabolism

Generally, cancer cells have the ability to proliferate from one abnormal cell to more than 10^9^ cells (the total number of cells ~1 cm in diameter in a tumor) if suitable conditions are available [20]. Tumor cells will modify their metabolism to guarantee survival, overpower host immune attack, and maintain the proliferative capacity to induce their lethal effects and sustain survival [21]. Cancer cells undergo physiological adaptations to preserve their survival under many stressful conditions, such as hypoxia and food hunger in order to fulfill their massive growth requirements. Such metabolic modifications generate abnormal metabolic events when compared to normal cells. This reprogrammed metabolism is seen as a cancer signature, as many metabolic modifications are predominant in several other types of cancers [22]. Similar to normal cells, cancer cells also have to produce ATP to maintain both daughter cells formed through division, rely on metabolic intermediates or biosynthesis by-products and, most critically, to compete with the oxidizing effects to mitigate the impact of reactive oxygen species (ROS). These altered metabolic and bioenergetic mechanisms, significantly elevated biosynthesis and redox equilibrium, are crucial to cancer progression [23]. Therefore, proliferating cells need to obtain high amounts of lipids, nucleotides, and amino acids. Through using by-products and derivatives of the TCA (tricarboxylic acid) cycle, cells can generate this biomass [24]. It would appear that fulfilling all these requirements would require a significant boost in glucose uptake in tumors. The high glucose consumption in many, but not all, tumors have been verified by PET (positron emission tomography) imaging and glucose consumption rates beyond the levels that can be easily explained by energy or metabolite requirements [25]. Moreover, the utilization of glutamine meets a symmetrical pattern of excessive consumption [26]. It is believed that cancer metabolism could be described as upregulation of the metabolism of both glucose and glutamine for the production of energy.

### 2.1. Glucose

Glucose is the principal source of cellular energy and in the presence of oxygen, it is metabolized to pyruvate through glycolysis, which is transported to the mitochondria, where it is oxidatively metabolized into CO_2_ in the tricarboxylic acid (TCA) cycle and the oxidative phosphorylation via electron transport chain (ETC) to produce high amounts of energy (for each molecule of glucose about 32 to 34 molecules of ATP are produced) [27]. Cells may also undergo anaerobic glycolysis without oxygen, i.e., fermentation, diverting the resulting pyruvate molecules towards lactate production which is less effective in ATP production than the TCA cycle coupled with oxidative phosphorylation. Similarly, cancer cells primarily metabolize glucose, but in contrast to normal cells and in spite of the presence of oxygen, glycolysis produces lactate leading to the generation of two ATP per molecule of glucose. Consequently, cancer cells need a high glucose utilization efficiency so as to fulfill their energy and anabolic requirements, Otto Heinrich Warburg in 1920 found that even in ample oxygen, glycolysis was improved in cancer cells. However, glycolysis was commonly known to increase under anaerobic conditions [28]. Despite deficiency in oxygen, increased glycolysis in cancer cells seemed to be a new phenomenon and was referred to as aerobic glycolysis or the “Warburg effect” [29]. Consequently, the high quantity of glucose catabolism into lactate has been the most widespread metabolic phenotype detected in cancer cells, contributing to the deposition of lactate by-products within the tumor’s microenvironment [20]. Currently, it is obvious that cancer cells experience aerobic glycolysis due to oncogene stimulation, tumor suppressor genes inhibition, and activation of phosphatidylinositol 3-kinase (PI3K) pathway, and that one benefit of high glycolytic frequencies is the availability of anabolic pathway substrates [30]. The expression of glucose transporters in tumor cells is deregulated by some oncogenes (such as *Myc* oncogene), resulting in over-expression of these transporters, especially GLUT1 and GLUT3 [31]. The idea that cancer cells utilize glucose as a fuel is explained as follows; glucose begins to be processed in the cytosol, and is converted to glucose-6-phosphate (G6P) by Hexokinase (HK). Glucose-6-phosphate (G6P) is the splitting point from glycolysis to the pentose phosphate pathway oxidative branch (PPP) that produces the ribose group needed for nucleotide synthesis (RNA and DNA synthesis). This pathway is important in order to enable cancer cells to fulfill their anabolic demands and survive oxidative and nutritional stress [32]. In this pathway, glucose-6-phosphate (G6P) is broken up into three-carbon compounds, 3-phosphoglyceraldehyde, which is the main point for the non-essential amino acid serine synthesis. Phosphoenolpyruvate (PEP) is also formed by glycolysis and end up producing two pyruvate molecules [20]. The glycolytic enzyme pyruvate kinase enzyme (PK) is the final rate-limiting enzyme and is essential in pyruvate and ATP production. This enzyme has four isoforms (M1, M2, L, and R) which are expressed in different cell types. The PKM2 isoform is highly expressed in tumors thus shifting glucose metabolism towards anabolism through aerobic glycolysis and allowing the pyruvate product in the cytosol to be reduced to lactate by lactate dehydrogenase enzyme (LDH) [33,34,35]. In cancer cells, lactate dehydrogenase enzyme (LDH) expression is upregulated which plays a crucial role in cancer characteristics [33].To sum up, cancer cells use catabolism of glucose as a main energy-generating mechanism by glycolysis and also from glucose, many biosynthetic compounds and NADPH molecules are produced.

### 2.2. Glutamine

**Figure 1:**
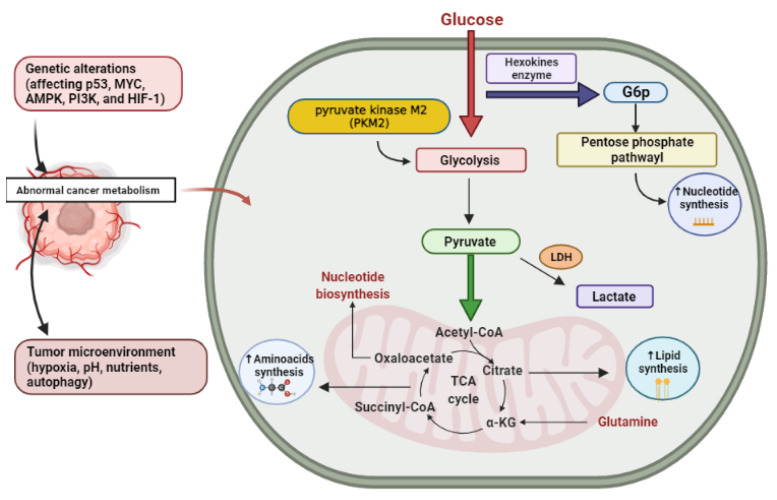
Cancer altered metabolism. G6P, glucose-6-phosphate; LDH, lactate dehydrogenase enzyme; α-KG, alpha-ketoglutarate. Created using Biorender software.

The most common circulating amino acid in humans is glutamine. Glutamine is also the second most abundant nutrient after glucose in vitro cell culture environments, and its intake surpasses protein synthesis requirements [26]. Via controlling mitochondrial reactive oxygen species (ROS) and proliferation, glutamine impacts the signaling pathways needed for cancer development, survival, and metabolism [36,37]. Glutamine is taken up by proliferating cancer cells and converted to glutamate by various deamidation and transamination reactions, especially mitochondrial amidohydrolase glutaminase [38]. Then, glutamate is transformed to alpha-ketoglutarate (α-KG) by glutamate dehydrogenase activity (or an aminotransferase) [39]. Moreover, glutamine is used by rapidly developing tumor cells as a source of carbon for energy production as well as for the regenerating intermediates of the TCA cycle such as pyruvic acid, oxaloacetate, and α-KG to compensate for the continuous depletion of citrate exported from the mitochondria for lipid synthesis [37]. (Figure 1) showing the metabolic alteration in cancer cell.

## 3. Diabetes Relationship with Metabolic Syndrome (MetS)

A metabolic syndrome was described for the first time by Reavan in 1988, in which he suggested that it was an essential feature in developing Chronic Heart disease (CHD) and Diabetes Mellitus type II (DMTII), mostly through insulin resistance [40]. Afterwards, established criteria for metabolic syndrome clinical identification have been emerged by NCEP-ATP III (the National Cholesterol Education Program/Adult Treatment Panel III) [41,42], WHO (the World Health Organization), IDF (the International Diabetes Federation) [43], and AACE (the American Association Clinical Endocrinologists) [44] (Shown in Table 1).

According to NHANES III (the 3rd National Health and Nutrition Examination Survey) criteria, almost 47 million people have metabolic syndrome [45]. Moreover, more than 2 in 10 cancers in the UK are thought to be related to metabolic syndrome [46]. The risk of developing metabolic syndrome is increased with age, and it has been estimated that, in the age group of over 50 years, more than 40% in the United States and nearly 30% in Europe of this age group have metabolic syndrome [47,48]. Recently, Esposito et al. using a meta-analysis, examined 38,940 patients with metabolic syndrome and cancer, and the study results showed that metabolic syndrome increases the risk of several cancers including colorectal, pancreas, and liver cancers. However, the reported results showed differences between genders. In men, the metabolic syndrome was strongly linked with both liver and colorectal cancers but weakly linked with bladder cancer. On the other hand in women, the metabolic syndrome was linked with endometrial, pancreas, breast (especially in postmenopausal women), colorectal, and ovary cancers [49]. Table 1 summarizes various definition of metabolic syndrome.

Insulin is considered the primary anabolic hormone and has an essential function in stimulating cell proliferation [51]. In healthy individuals, the blood glucose level is tightly controlled by both insulin release and insulin-mediated glucose uptake in the tissues [52]. On the other hand, when the response of the normal cells to insulin is weakened, a condition called insulin resistance is produced. As a result, the pancreas β-cells respond to this condition by increasing the secretion of insulin into the circulation, causing hyperinsulinemia (High insulin concentration in the blood] in order to maintain a normal blood glucose level [53]. Insulin resistance creates a favorable environment for neoplastic tissue survival and cancer cell development [54,55,56], this could be explained through the abnormally elevated levels of growth factors, ROS (Reactive Oxygen Species), adipokines, and proinflammatory cytokines that are associated with this condition.

The primary mechanism for many features of metabolic syndrome is suggested to be insulin resistance or hyperinsulinemia. Studies have shown that chronic hyperinsulinemia is also associated with several cancer types such as colorectal, pancreatic, endometrial, and breast cancers [57]. Hyperinsulinemia reduces the production of IGFBP-I and -II (Insulin-like growth factor binding protein) thus increasing IGF-I (Insulin-like growth factor) level in the blood, and promoting tumor development by changing the cellular environment [58]. In addition, hyperinsulinemia inhibits the synthesis of SHBG [Sex hormone-binding Globulin] leading to increased free sex hormone levels thus promoting sex hormone-dependent cancers such as endometrial, breast and prostate cancers [58]. Metabolic syndrome is also characterized by hyperglycemia [29], however, this does not mean that every diabetic patient has MetS since it is about three times more common than diabetes [59]. The results of a study done by Flood et al. to investigate the relationship between insulin, fasting glucose, and colorectal adenomas recurrence risk, showed that the risk for colorectal recurrence is higher in patients with elevated insulin and glucose [60].

A relationship between insulin resistance or hyperinsulinemia with different types of epithelial cancers has been shown by several population-based studies. Initial studies on prostate cancers showed a link with plasma IGF-I concentration [61]. On the other hand, different studies have confirmed that both elevated IGF-I and insulin levels are associated with prostate cancer risk, prospectively [62,63,64]. Moreover, a link has been shown between hyperinsulinemia and breast cancer risk (measured by fasting C-peptide concentration) mainly in postmenopausal breast cancer [65] and also with endometrial cancer risk independent of estradiol [66].

## 4. Metabolic Link between Diabetes and Cancer

The potential metabolic links between DM and cancer development are hyperinsulinemia, hyperglycemia, and chronic inflammation due to fat imbalanced metabolism.

### 4.1. Hyperinsulinemia

The role of insulin’s in carcinogenesis is mainly influenced by insulin receptor (IR), Insulin-like Growth Factor (IGF), and Insulin-like Growth Factor Receptor (IGF-R) [4]. In diverse human malignancies, it was found that there is overexpression in the IR, the fetal isoform IR-A in particular, and in the IGF-R [67,68]. Moreover, tumor cells can produce hybrid forms of insulin and IGF-1 receptors that can either be activated by insulin, IGF-1, and/or IGF-2 [69]. Insulin receptor (IR) is composed of two α-subunits and two β-subunits, expresses the activity of tyrosine kinase and is strongly activated by insulin, while both IGF-1 and IGF-2 possess weaker activation [70,71]. The biochemical processes of insulin signaling proceed via autophosphorylation of IR itself and its direct substrate followed by activation of several pathways including the lipid kinase phosphor-inositide-phosphoinositide 3-kinase (PI3K), protein kinase B (Akt), and mammalian target of the rapamycin signaling pathway (mTOR) eventually leading to carcinogenesis via abnormal cells proliferation and inhibition of apoptosis [72]. Increased mitogenesis was also observed in activated IRs in an insulin-resistant environment [73]. Due to the structural similarity between IGF-R and IR (approximately 60% homology), insulin may also activate IGF-R [73]. Activation of IR produces mainly metabolic effects, on the contrary activation of IGF-R by insulin produces mitogenic effects including cell proliferation, angiogenesis, and metastasis [67,74]. Studies have verified that restriction of calorie consumption leads to reduced levels of insulin and IGF-1, complemented by declined insulin resistance and repressed mTOR pathway [75,76]. Furthermore, hyperinsulinemia stimulates an increase of IGF-1 level through hepatic activation of growth hormone receptor (GHR), resulting in increased secretion of growth hormone (GH) which stimulates IGF-1 [77]. An elevated level of IGF-1 is considered a predisposing factor for premenopausal breast, prostate and colorectal carcinogenesis [78]. Additionally, hyperinsulinemia also leads to a decreased level of IGF-binding protein 1,2, and 3 resulting in an increased level of bioavailable IGF-1 [67,79]. Elevated insulin level also minimizes liver production of sex-hormone-binding-protein (SHBG) and shortage of this protein increases the level of bioavailable estrogen or testosterone thus increasing the risk of hormone-dependent cancers [80]. Likewise, hyperinsulinemia enhances the production of leptin (the mitogenic adipokine) by hypoxia-inducible factor-1α (HIF-1α). Although the mitogenic effect of leptin was believed to stimulate proliferation of esophagus, breast, and prostate cancer cells; it showed inhibitory activity on pancreatic cancer cells [81].

### 4.2. Hyperglycemia

One of the notable characteristics of cancer cells is the increased uptake of glucose and enhanced glucose metabolism [82] to be able to grow and divide rapidly. Cancer cells possess altered metabolism via aerobic glycolysis, namely the “Warburg effect” [29]. Hyperglycemia promotes tumor growth via proliferation, anti-apoptosis, and metastatic activity [83]. The proliferative effect induced by hyperglycemia occurs as a result of overexpression of the following: glucose receptors (GLUT-1, GLUT-3) in tumor cells, protein kinase C (PKC-α), peroxisome proliferator-activated receptor (PPAR α and γ), and epithelial growth factor (EGF) [83]. On the other hand, the anti-apoptotic activity of hyperglycemia is a consequence of the decreased level of prolyl hydroxylase (PDH) enzymes that eliminate the hypoxia-inducible factor α (HIF-α), thus leading to elevated HIF-α, which is a vital regulator of tumor cell survival in a hypoxic and anabolic environment. Additionally, hyperglycemia preserves cytochrome-c mediated apoptosis by elevated nicotinamide adenine dinucleotide phosphate (NADPH). Hyperglycemia also promotes metastasis and invasiveness due to several factors including epithelial to mesenchymal transition (ETM) process, oxidative stress via reduction of reactive oxygen species (ROS) and increased superoxide dismutase (SOD), and elevated expression of zinc transporters leading to enhanced zinc absorption which is involved in migration of cancer cells [84]. Moreover, oxidative stress is a possible risk factor for carcinogenesis in diabetic people since it is the initial mechanism of insulin resistance; which is due to the inactivation of insulin receptors resulting from the overproduction of superoxide in the mitochondria [72].

In addition, increased glucose levels affect the epigenetic regulation resulting in what is known as “hyperglycemic memory”; a condition that allows the activation of oncogenic pathways in hyperglycemia-exposed tumor cells, even after constant glucose level normalization [85]. Glucose-induced constant expression of NF-κB p65-gene is the probable reason behind this mechanism [85]. Interestingly Jee et al. suggested that fasting glucose level (FGL) > 125 mg/dL increases the risk of cancer incidence [86]. On the contrary, a meta-analysis of major published trials clarified that the risk of cancer development was not reduced in type 2 diabetics who followed an intensified glycemic control [87].

### 4.3. Chronic Inflammation Due to Fat Imbalanced Metabolism

**Figure 2:**
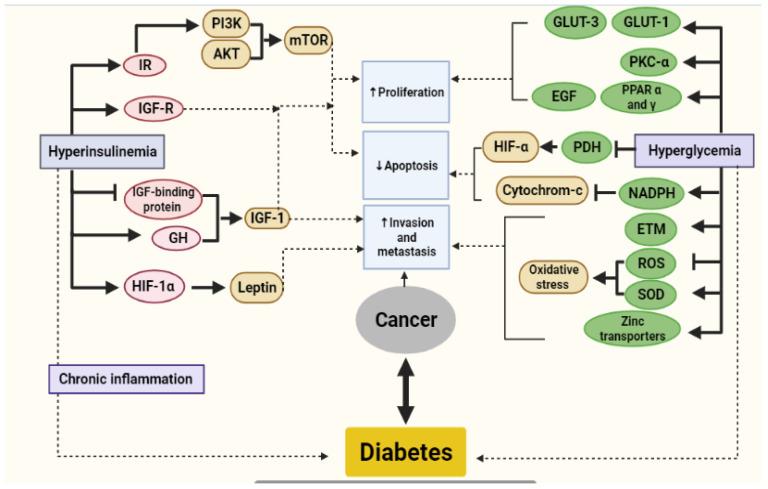
Metabolic link between diabetes and cancer. (→), activation; (┬), inhibition; IR, insulin receptor; IGF-R, insulin-like growth factor receptor; PI3K, phosphoinositide 3-kinase; AKT, protein kinase B; mTOR, rapamycin signaling pathway; GH, growth hormone; IGF-1, insulin-like growth factor 1; HIF-α, hypoxia-inducible factor-1α; GLUT, glucose receptor; PKC-α, protein kinase C; PPAR, peroxisome proliferator-activated receptor; EGF, epithelial growth factor; PDH, prolyl hydroxylase; NADPH, nicotinamide adenine dinucleotide phosphate; ETM, epithelial to mesenchymal trasition; ROS, reactive oxygen species; SOD, superoxide dismutase. Created using Biorender software.

Obesity promotes tumorigenesis according to evolving studies [88,89] stressing that almost 80% of T2DM cases are associated with obesity and overweight [90]. Numerous studies have also suggested that excess body adipose tissue is a risk factor for various types of cancers, including breast, endometrial, esophageal, pancreatic, and colorectal, cancers [88]. The tumor-promoting activity of obesity can be probably explained by mechanisms such as alteration in sex hormones metabolism, changes in the levels of adipokines, chronic inflammation, and insulin resistance [80,88]. Moreover, metabolic and mitogenic variations induced by excessive adipose tissue enhance the impact of hyperglycemia and hyperinsulinemia observed in T2DM. Figure 2 showed the metabolic relationship between diabetes and cancer.

### 4.4. Correlation between DM and Specific Cancer Examples

#### 4.4.1. Liver Cancer

The most frequently observed malignant liver neoplasm in patients with DM is hepatocellular carcinoma (HCC) [91]. The association between DM and a higher risk of HCC development was initially reported by Lawson in 1986 [92], and was effectively supported by further researchers [91,93,94]. These studies found that HCC is highly diagnosed in patients with non-alcoholic fatty liver disease (NAFLD), in obese patients with insulin resistance, and in T2DM patients [95,96]. These disease conditions promote hepatic oncogenesis by several mechanisms, such as insulin resistance, modified adipokines pathophysiology, lipotoxicity and oxidative stress in the form of systemic inflammation. Furthermore, obesity accompanied by insulin resistance causes a decrease in adiponectin concentration and an increase in leptin, TNF-α, IL-6, and free fatty acids (FFA) concentrations, promoting growth, multiplication, and carcinogenesis in hepatic cells [96].

Moreover, the liver is subjected to high insulin concentration as a result of portal circulation, thus leading to insulin-induced proliferation and suppression of apoptosis in hepatic cells [97].

Additionally, HCC cells exhibit overexpression of both IGF-1 and insulin receptor substrate-1 (IRS-1), leading to the magnification of insulin effect. Furthermore, IRS-1 activates PI3K signaling and prevents apoptosis regulated by Transforming Growth Factor-β1 (TGF-β1), which is essential in hepatic cell carcinogenesis. Studies have also proposed that both T1DM and T2DM predispose to HCC, probably via hyperglycemia; in particular, a slightly stronger correlation was noted with T1DM than that with T2DM [98]. On the other hand, data from various studies have suggested that T2DM with its simultaneous metabolic disorders activate oncogenesis in hepatic cells [99,100].

#### 4.4.2. Prostate Cancer

Prostate cancer (PC) is the only described cancer in the literature associated with DM [101]. Several studies reported the significant converse association between PC and DM which suggested a protective effect of DM against PC [101,102,103]. Potential causes of this association involve a low testosterone level (probably due to high glucose level) and hypo-insulinemia observed in T1DM or T2DM in the long term. On the contrary, Li et al. suggested that DM is associated with an improved risk of advanced PC development [104].

## 5. Impact of Diabetes and Obesity on Cancer

Obesity is a major public health concern around the world, and it is now considered a crisis. It occurs as a result of changes in lifestyle (lack of physical activity, a high fat/calorie diet, a high carbohydrate diet) and is often related to lifestyle factors such as cigarette smoking and alcohol consumption. According to World Health Organization (WHO) over 1.9 billion people aged 18 and up were overweight in 2016. Over 650 million people were overweight (body mass index ≥ 25 kg/m^2^) or obese (body mass index ≥ 30 kg/m^2^) [105]. Obesity and type 2 diabetes, “Diabesity is a new concept for diabetes that affects people who are obese” have become much more common across the world, which are commonly related to metabolic disorders and both are linked to a higher incidence and mortality rate which can accelerate cancer progression [106]. Diabesity has been associated with the development of pancreatic cancer and its pathogenesis in many studies [107]. In addition, it contributes to an increased risk of a number of different cancers, including breast, hematological and prostate cancer [108,109,110]. Diabesity has been related to cancer progression through a number of mechanisms, including 1. insulin-like growth factor signaling, activation of the insulin/IGF signaling pathway has been presumed to lead to tumor initiation and/or progression just at the cellular level via tumor cell-specific mechanisms such as cell division activation and glucose metabolism [111] and epithelial-to-mesenchymal transition (EMT) [110] 2. Insulin resistance and hyperinsulinemia are common in individuals with diabesity, C-peptide levels as indicators of insulin secretion have been tested in many studies to see whether hyperinsulinemia is related to cancer risk and mortality [112]. A 37% increased risk of colorectal cancer has been linked to elevated C-peptide levels [113]. According to the Physicians Health Report, obese men with increased C-peptide levels are likely to die from prostate cancer four times more than men with normal C-peptide levels [114]. 3. Adipose tissue factors, adipose tissue is an important organ for the production of adipokines, inflammatory cytokines, and enzymes that are dysregulated in obesity and type 2 diabetes and potentially contribute to tumor growth and metastases [106,107,110]. 4. Gut microbiome, Changes in the gut microbiota have been linked to an increased risk of some cancers by inducing inflammatory responses and leading to cancer proliferation [109]. Recognizing the link between adipose tissue and tumors, the impact of obesity on immune cell function, and the effect of metabolic health on cancer progression will be crucial for enhancing current therapy responses and designing new therapies that address systemic metabolic.

## 6. Therapeutic Challenges in Treating Patients with Diabetes and Cancer

Various studies have indicated that cancer patients with diabetes have an inferior prognosis in comparison to non-diabetic patients. This could be explained since diabetes in cancer patients is a reason for higher infection rates, shorter remission periods, and higher mortality rates [55,115,116,117]. Consequently, a variety of challenges face clinicians when treating cancer patients with diabetes because of the cardiac, neurologic, and renal complications associated with diabetic patients. Chemotherapeutic choices and treatment guidelines and, eventually, cancer outcomes could be affected due to the avoidance of using some of the best clinical agents with the best survival rates in cancer patients since these therapeutic agents may cause other disease complications [118].

### 6.1. Challenges Using Chemotherapeutic Agents

Cardiac, renal, and neuropathic complications usually develop in patients with poorly controlled diabetes and these complications can also be caused or exacerbated by many chemotherapeutic treatments. Anthracyclines, for example, may cause cardiotoxicity [119,120], and cisplatin could cause renal insufficiency. Consequently, the inability to use cisplatin in testicular cancer therapy decreases the overall response rates and survival [121]. In addition, neurotoxicity, which is peripheral sensory neuropathy that occurs approximately in (40–50%) of diabetic patients, mainly in the feet, may also be caused by the use of cisplatin, vincristine, and paclitaxel; unfortunately, these side effects are usually permanent [122,123]. Moreover, in order to achieve a successful cancer treatment, at least 85% of the chemotherapeutic dose should be given which necessitates careful monitoring of patients with cancer and diabetes before starting and during chemotherapy. Treatment of such patients must depend on the patient’s clinical picture since any alterations of the patient’s dose, time of administration, or changing between chemotherapeutic agents may affect the outcomes and lower the treatment response rate and also shorten patient’s survival rates [117,124,125].

### 6.2. Challenges Using Glucocorticoids

Several cancer treatment protocols regularly use glucocorticoids either in high doses for short-term therapy or in lower doses to alleviate nausea and vomiting that accompany treatment with chemotherapeutic agents. Therefore, before starting glucocorticoid therapy in any cancer patient, they should be tested for diabetes and also monitored closely after that, mostly because these patients have a high chance of being undiagnosed since up to a third of diabetic persons in the general public are undiagnosed [117]. The mechanism of glucocorticoid action in raising blood glucose levels is by increased insulin resistance, glycogenolysis, gluconeogenesis, and decreased insulin production and secretion [126]. Diagnosing cancer patients with diabetes during receiving glucocorticoid therapy is a common phenomenon as well. In addition, the most important factors for glucocorticoid-induced diabetes are increased age, obesity, a family history of diabetes or a history of gestational diabetes and treatment with high doses of steroids [127].

### 6.3. Challenges of Using Cancer Treatments While on Glucose-Lowering Treatments

Glucose-lowering treatments may influence cancer treatments and ultimately impact cancer-specific mortality. A retrospective study was carried out by MD Anderson Cancer Centre in order to determine whether treatment with metformin had affected the pathologic complete response (pCR) rates in females with both diabetes and breast cancer on treatment with neoadjuvant chemotherapy [128]. The study showed that the rate of pCR was 24% for women on metformin treatment, 8.0% for women on therapy other than metformin, and 16% for non-diabetic women; those results highlight that many diabetic patients manifest a weakened response rate to chemotherapy in comparison with non-diabetic patients. Furthermore, another two recently published studies affirmed similar results when metformin was used by diabetic patients with colorectal cancer [129], and also patients with advanced non-small-cell lung cancer undergoing first-line chemotherapy [130].

## 7. Suggested Therapies for Cancer and Diabetes Patients

It is essential to state that glucose may be a potentially relevant cancer mediator taking into consideration the complexity of interactions between diabetes, diabetes treatments, and cancer. The Warburg hypothesis emphasizes how many types of cancer depend on glycolysis for energy, i.e., “glucose addiction” [29].

One of the most effective diabetes management is to maintain improved control over blood glucose, thus reducing the risks of developing the complications associated with diabetes which leads to the minimization of morbidity and mortality rates. Therefore, regarding glucocorticoid therapy, rather than using a single bolus dose of steroids, giving steroids intravenous over 24 h or multiple doses during the day can better help control hyperglycemia in these patients. Diabetic patients treated with oral hypoglycemic medications may be kept on their medications while carefully monitoring their glucose levels. Nevertheless, in this case, these medications are usually inadequate for controlling hyperglycemia. On the other hand, diabetic patients who are on insulin therapy before starting the glucocorticoid therapy will usually need both basal and preprandial insulin administration and would need to increase their usual dose(s) of insulin two to three times. Moreover, in order to manage a patient’s induced or exacerbated hyperglycemia when using steroids, insulin is the preferred method in patients known with diabetes [126,127,131], and insulin doses are determined depending on the patient’s weight. A faster way to lower blood glucose levels is by intravenous insulin infusion which will also estimate the total required daily insulin dose for insulin naïve patients. However, many hospitals do not allow the use of intravenous insulin drips unless it is used in intensive care units due to the risk of hypoglycemia. Finally, insulin doses should be titrated daily and should also be tapered whenever glucocorticoid therapy is tapered in order to avoid hypoglycemia [126,127,131,132,133,134].

In addition, clinicians often need a flexible approach to diabetes management in cancer patients because of the cyclical nature of chemotherapeutic agents, since platinum-based chemotherapy (e.g., cisplatin), mTOR kinase inhibitors (e.g., everolimus), and ABL kinase inhibitors (e.g., nilotinib) have all been connected to hyperglycemia.

Unfortunately, there are few published articles related to this area. However, from the author’s experience, it is well known that diabetic patients with cancer need to be monitored frequently for hyperglycemic during therapy, especially in cases of advanced or incurable cancer patients undergoing palliative care [135].

## 8. Natural Products Targeting Diabetes and Cancer

### 8.1. Resveratrol

Resveratrol is a natural polyphenol that belongs to the class of stilbene [136]. It is derived from various plant types and is presented in 34 families containing 100 species [137]. In peanuts, soybeans, purple grapes and pomegranates, high resveratrol concentrations have been found [138]. Resveratrol (3,5,40-trihydroxystilbene) is a stilbenoid and phytoalexin generated by a variety of plants as a result of injury or other pathogenic attacks [139]. Resveratrol has historically been used to treat stomach pain, hepatitis, arthritis, urinary tract infections, and inflammatory and cardiovascular diseases [140]. Several studies have recently reported the anticancer and antidiabetic effects of resveratrol.

Resveratrol demonstrated antiproliferative and apoptotic effects on human cervical carcinoma cells by stimulating caspase-3 and caspase-9, impeding cell growth, inducing p53 expression, and upregulating the X-associated protein Bcl-2 [141]. It prevented the proliferation of colon cancer cells and induced cell apoptosis by suppressing the signaling pathway of AKT/STAT3 [142]. It enhanced paclitaxel’s apoptotic and oxidant effects by stimulating the TRPM2 channel in glioblastoma cells [143]. Additionally, it inhibited pancreatic cancer cell metastasis by controlling the expressions of vimentin, CTA-2, IL-1β, TNF- α, and N-cadherin [144]. Zhao et al. indicated that encapsulated resveratrol within peptide liposomes decreased free resveratrol’s toxicity and enhanced the physicochemical properties. It stimulated apoptosis in breast cancer by rising Bcl-2 activity, controlling p53 and Bax expression, and inducing the activation of caspase-3 [145]. A combination of thymoquinone and resveratrol was studied in both in vivo and in vitro models, and the findings demonstrated substantial cancer cell inhibition, angiogenesis suppression, and apoptosis elevation [146,147]. A synergistic effect between resveratrol and doxorubicin against breast cancer cells was observed. In Ehrlich ascetic carcinoma cells carrying mice, combination therapy enhanced life span and decreased tumor volume [148].

Resveratrol was examined in mice by analyzing postprandial glucose concentrations for its ability to inhibit α-glucosidase. The results showed that resveratrol delayed carbohydrate absorption, resulting in a reduced post-prandial blood glucose response [149]. Several studies showed the antidiabetic effect of resveratrol [150]. Resveratrol was revealed to enhance muscle and liver glucose absorption, decrease adipose tissue and liver inflammation, increase adaptive thermogenesis capability, and inhibit pancreatic insulin secretion [149]. Quercetin and resveratrol were considered to have positive effects on diabetes. A study was conducted to investigate the combined antidiabetic action of quercetin and resveratrol in diabetic rats induced by streptozotocin. Elevated serum blood glucose, insulin levels, and dyslipidemia were significantly improved in diabetic rats by quercetin, resveratrol, and combination therapies. These compounds inhibited oxidative stress and tissue injury biomarkers substantially. Co-treatment of quercetin and resveratrol was useful against diabetes as it maintained the hepatic glucose metabolic enzymes activities and structure of pancreatic β-cells from the diabetes [151].

### 8.2. Curcumin

One of three components of phenolic difrauloylmethane compounds known as curcuminoids is curcumin. It is a major active ingredient found in Curcuma longa dried rhizomes (family: Zingiberaceae), generally referred to as turmeric [152,153,154]. Curcumin has several pharmacological properties such as anticancer, anti-diabetic, antioxidant, antiviral, antibacterial, anti-inflammatory, and wound-healing ability [152,154,155,156,157]. Curcumin has been recognized as an anticancer agent via various mechanisms of action, including cancer cell apoptosis activation, cancer cell metastasis suppression, and cancer cell growth inhibition [155,158,159,160]. Curcumin has been identified in several signaling pathways such as activation of apoptotic ligand-inducing tumor-necrosis-factor-related apoptosis (TRAIL) pathways in HT-29 and HCT-116 colon cancer cells by upregulating death receptor factor 5 [161]. In HT-29 colon cancer, curcumin began Fas-mediated apoptotic pathway through caspase 8 activation [162]. Bax expression was upregulated and Bcl-2 was inhibited via phosphorylation at Ser15 and p53 activation in HT-29 colon adenocarcinoma cells [163], and HCT-116 [155]. The anti-cancer effect of curcumin on osteosarcoma was reported by inhibition of MG-63 cell proliferation and migration and inactivation of JAK/STAT signal [164]. Curcumin was also useful against prostate cancer as it interfered with nuclear factor κ (NFκB), epidermal growth factor receptor (EGFR), and mitogen-activated protein kinase (MAPK) [165].

A study revealed that 14 days treatment of human adipocytes with turmeric ethanol extract, containing curcumin, demethoxycurcumin, bisdemethoxycurcumin and ar-turmerone, led to improved adipocyte differentiation substantially in a dose-dependent manner, increase glucose elevation levels and enhance the activity of human peroxisome proliferator-activated receptor (PPAR)-gamma ligand-binding [166]. Furthermore, treatment of 3T3-L1 adipocytes with curcumin decreased glucose uptake, leptin levels, NF-kB p65 nuclear fraction, phospho-JNK1/2, phospho-IRS-1(S), MMP-2, MMP-9 and VEGF protein. However, it elevated insulin sensitivity, adiponectin levels, and IRS-2 protein [166].

Curcumin supplementation greatly stopped the progression and disruption of the renal lesion. It decreased the levels of urinary enzymes (acid phosphatase, alkaline phosphatase (ALP), aspartate aminotransferase (AST), and alanine aminotransferase (ALT)). Additionally, it decreased lactate dehydrogenase and renal glucose-6-phosphatase and increased ATPase activity [167]. The co-administration of curcumin and metformin reduced JAK/STAT signalling pathway expression substantially to suppress myocardial degeneration and diminish lipid peroxidation levels, IL-6, creatine kinase-MB (CK-MB), troponin I, and tumor growth factor-β1 (TGF-β1) [168].

### 8.3. Thymoquinone

The major phytochemical bioactive constituent identified in volatile oil isolated from *Nigella sativa* (black cumin, black seed) is thymoquinone, which has been used in several countries as a traditional medicine [146,169,170]. Thymoquinone has antihistaminic, immunomodulatory, antioxidant, antitumor, anti-inflammatory, and antimicrobial activities [170,171,172,173,174].

Thymoquinone can be used as an anticancer agent as it can alter the regulation of cell cycle, growth factor, apoptosis, tumor-suppressor gene, protein kinase enzyme, transcription factors, survival signals, and phase I and II enzymes [170]. Altering the progression of the cell cycle is an essential step in inhibiting the development and progression of cancer. Fatty acid-conjugated thymoquinone has a promising effect on apoptosis, cell proliferation, and signaling pathways [170]. In the human mammary breast cancer epithelial cell line, MCF-7, thymoquinone induced cell arrest at various stages according to the concentration used (25 and 50 μM) in vivo [175]. Furthermore, it was used to reduce serum TNF- α, IL-6, and iNOS enzyme production and improve histopathological outputs in Wistar rats with methotrexate-induced hepatorenal system injury [176]. It played an important role in suppressing endothelial cell migration and tumor angiogenesis. In HCT 116 human colon cancer cells, thymoquinone significantly reduced the phosphorylation of EGFR to tyrosine-1173 residues and JAK2 in vitro [177]. It has an antiproliferative effect, particularly when combined with doxorubicin and 5-fluorouracil, resulting in increased cytotoxicity in the xenograft mouse model of breast cancer [178]. A study showed that thymoquinone activates apoptosis by reducing antiapoptotic protein expression [179].

Thymoquinone has antidiabetic and antioxidant activities. It can reduce blood glucose level, triglycerides and cholesterol concentrations. On the other hand, it can improve the high-density lipoprotein, insulin sensitivity, and pancreatic β-cell regeneration resulting in substantial enhancement of the oral glucose tolerance test [180]. Glycolysis and Kreb’s cycle pathways were used by thymoquinone to enhance glucose utilization while gluconeogenesis was used to decrease glucose production [181]. Thymoquinone enhanced the antidiabetic effect of metformin [182]. It can maintain the integrity of pancreatic β-cells by enhancing oxidative stress which subsequently raises the level of insulin [181]. *Nigella sativa* consumption can reduce inflammatory and oxidative stress markers [183]. Serum high-sensitivity C-reactive protein, tumor necrosis factor-α, and malondialdehyde levels were significantly reduced by *Nigella sativa* intake, while total antioxidant ability and superoxide dismutase levels were significantly increased [183].

### 8.4. EGCG (Epigallocatechin Gallate)

The natural polyphenol that belongs to the class of flavonols is epigallocatechin-3-gallate (EGCG) [184]. Green tea (*Camellia sinensis, Theaceae*) [185] and cocoa products [186] are the major dietary sources of EGCG. Green tea was utilized in the past as a diuretic, astringent, stimulant, and to improve heart health in Indian and Chines medicine [187]. EGCG has numerous medical benefits, including suppression of tumor growth, inhibition of irregular blood clot formation, and reduction of LDL cholesterol levels [188]. EGCG is the most effective anti-inflammatory and anticancer agent among the various green tea catechin derivatives [189].

EGCG can be utilized as an anticancer agent as it has antimetastasis, antiproliferative, and pro-apoptosis activities. The antimetastasis activity of EGCG was mediated by suppression of MMP-2 protein expression through modulation of the Src signaling pathway [190]. The synergistic chemotherapeutic potential was demonstrated by the combination of EGCG with eugenol or amarogentin in the cervical cancer cell line [191]. The antiproliferative effect of EGCG was proved by its capability to suppress cyclinD1 and improve LIMD1, RBSP3, and p16 cell-cycle inhibitors at the G1/S cell cycle level [192]. Moreover, by reducing multi-drug resistance 1 signaling and stimulating the AKT/STAT3 pathway, EGCG was capable to alert cisplatin-resistant oral cancer CAR cell apoptosis and autophagy [193]. The effect of EGCG on doxorubicin-induced cytotoxicity of oral keratinocytes and anticancer activity against oral cancer cells was reported. It reduced the doxorubicin cytotoxic effect without impairing its anticancer efficacy [194].

EGCG has an antidiabetic effect. In a reversible and non-competitive way, EGCG inhibited α-glucosidase because of the complex formation between EGCG and α-glucosidase, where the hydrogen bonds played an important role, EGCG quenched α-glucosidase fluorescence. EGCG strongly affected the secondary structure and the microenvironment of α-glucosidase. Furthermore, EGCG increased the glucose uptake and stimulated GLUT4 translocation to the plasma membrane through PI3K/AKT signaling pathway in L6 skeletal muscle cells [195].

EGCG also has an antiobesity effect. Green tea supplementation can help obese patients lose weight and lower their body mass index by several mechanisms, including inhibition of ghrelin secretion and adipogenesis, improvement of adiponectin levels, and reduction of nutrient absorption [196].

### 8.5. Allicin

Allicin is a sulfenic acid thioester, or allyl thiosulfinate. It is predominantly present in garlic (*Allium sativum*) and belongs to the family Liliacerae [197]. Allicin has various biological activities, including anticancer, antidiabetic, antimicrobial, and anti-inflammatory activity. Allicin substantially inhibited cholangiocarcinoma cell invasion and cell proliferation. It prohibited cell migration and prompted apoptosis via upregulation of SHP-1 and inhibition of the activation of STAT3 [198]. A study showed that allicin improved the X-ray radiotherapy sensitivity in colorectal cancer by inhibiting the signaling pathway of NF-κ [199]. Furthermore, allicin demonstrated anti-tumor activity against HCMV-infected glioma cells through cytokine release downregulation, p53 activity stimulation, and radiotherapy sensitivity enhancement [200]. Another study revealed allicin activity in stimulating apoptosis of cells and in suppressing ornithine decarboxylase which is a rate-limiting enzyme in neuroblastoma cell proliferation [201]. By increasing cyclin D1 and reducing MMP-9 mRNA expression, allicin inhibited melanoma cell growth [202]. Allicin also has synergistic anticancer activity against colorectal and lung carcinoma cells with 5-fluorouracil [203].

Allicin has antidiabetic activities. It was helpful in the treatment of diabetic nephropathy [204]. It enhanced insulin levels, reduced hyperglycemia, and prohibited changes in (GLUT4) and IRSs expression induced by diabetes [204]. It also increased the expression of Nrf2 and decreased the expression of Keap1, HIF-1α, SBP, and VEGF [204]. Treatment with allicin successfully decreased autoantibodies and anti-islet cell antibodies (ICA) for type 1 diabetes (IDDM). As a result, pancreatic tissues were restored and the decreased serum insulin level was greatly increased [205].

### 8.6. Emodin

Emodin is most widely isolated from the *Rheum palmatum* (Chinese rhubarb, family: Polygonaceae) roots and rhizomes. It is also existed in *Polygonum multiflorum* (Chinese knotweed), *Polygonum cuspidatum* (Asian knotweed), *Cassia obtusifolia* (Chinese senna, family: Fabaceae), *Aloe vera* (family: Asphodelaceae), *Aspergillus wentii,* and *Aspergillus ochraceus* [206,207,208,209]. Emodin (1,3,8-trihydroxy-6-methyl-anthraquinone) is a natural derivative of anthraquinone [210]. It has different Pharmacological activities, including antitumor, antimetastatic, antiproliferative, antidiabetic, antiviral, antibacterial, anti-inflammatory, and immunosuppressive activity [211,212,213,214]. Emodin’s molecular mechanisms are apoptosis, cell cycle arrest, and expression promotion of the detoxification enzymes hypoxia-inducible factor 1α, glutathione phase I and II, and glutathione S-transferase P,N-acetyltransferase. Emodin can also inhibit angiogenesis, invasion, migration, and formation of chemical-induced carcinogen-DNA adducts, HER2/neu, CKII kinase, and p34cdc2 kinase human cancer cells [215]. Tumor-associated angiogenesis was inhibited via the reduction of ERK phosphorylation.

Emodin has an important role in the treatment of diabetic nephropathy. It can reduce hyperglycaemia and stimulate cell proliferation and fibronectin expression by preventing cellular FLICE-inhibitory protein (cFLIP) and p38MAPK pathway. Additionally, it has a PPARγ-activating effect. It improved the symptoms of diabetic animals through PPARγ pathway regulation. Furthermore, glycolysis was improved by emodin through the AMPK signalling pathway [216].

### 8.7. Genistein

Genistein [40,5,7-trihydroxyisoflavone or 5,7-dihydroxy-3-(4-hydroxyphenyl) chromen-4-one] is an isoflavonoid derivative and it belongs to phytoestrogen classes. It exists in glycosylated or free forms in food (mainly legumes). It was firstly isolated from *Genista tinctoria.* It was also found in *lycine max* (Soybean), soy-based foods, soy-based drinks, Lupin (*Lupinus perennis*), broad beans and chick peas. Genistein has pharmacological activities, including anticancer, antidiabetic, antiosteoporetic, and estrogenic activity [217,218]. The mechanisms of genistein as anticancer agent include reducing proliferation, preventing angiogenesis and metastasis, and stimulating apoptosis [219,220]. Genistein has a good inhibitory effect against various cancer cell lines, including melanoma (MML-1 and SK-MEL-2), breast carcinoma (MDA-MB-231 and T47D), pancreas carcinoma (BxPC-3 and PANC-1), colon carcinoma (HT29 and COLO201), glioblastoma (U87 and LN229), and lung carcinoma (A549 and NCI-H460) [221]. Genistein was documented to inhibit cyclooxygenase-2 (COX-2) directly and indirectly by suppressing COX-2-stimulating factors such as activated protein-1 (AP-1) and Nf-κB. In pancreatic, colon, breast, and lung cancers, COX-2 overexpression was recognized and its inhibition was associated with decreased cancerous tumor growth in the esophagus and colon [217].

A study stated that genistein has anti-inflammatory and antidiabetic functions, especially direct effects on the proliferation of β-cells and the secretion of insulin. Genistein improved glucose homeostasis [222]. In β-cells, phytonutrient genistein easily stimulated cAMP signaling. It also improved the mass of islets in diabetic mice. CAMP generation genistein stimulation was eliminated in islets exposed to G15 unique GPR30 inhibitor or GPR30 deficient islets (GPR30−/−) mice [223].

### 8.8. Parthenolide

In the Asterceae family of medicinal plants, especially in *Tanacetum parthenium* (feverfew) [224], parthenolide is an essential naturally occurring metabolite [225]. It can also be found in *Tanacetum vulgare* (tansy) and *Tanacetum larvatum* [226]. Parthenolide was traditionally utilized to treat rheumatoid arthritis, fever, and migraine, while lately, it has been shown to have anticancer effects in various types of cancer, including bladder cancer, breast cancer, cholangiocarcinoma, pancreatic cancer, leukemia, prostate cancer, and cholangiocarcinoma [227]. Parthenolide’s anticancer mechanisms include DNA replication inhibition [216], STAT3 inhibition, apoptotic pathway stimulation [217], p.53 activation, and upregulation the production of reactive oxygen species (ROS) [228]. A study reported that parthenolide directly affected malignant cells without affecting normal cells [229]. Parthenolide was effective against distinct cholangiocarcinoma cell lines [230], because it induced apoptosis. Parthenolide inhibited breast cancer stem-like cells by stimulating oxidative stress and necrosis [231]. Furthermore, the growth of transplanted glioblastoma cells was significantly inhibited by parthenolide [232]. In pancreatic cancer cells, parthenolide can improve gemcitabine’s antiproliferative effects [233].

Parthenolide can reduce inflammation and remodel the impaired insulin signaling pathway, allowing cubilin and albumin uptake to be expressed [234]. During adipogenesis, adipogenic factors and lipid accumulation were reduced by parthenolide. In a dose-dependent manner, parthenolide inhibited adipogenic factors (PPARc and C/EBPa) and its target protein FABP4 production [235]. The regulation of reactive oxygen species production by parthenolide was linked to the control of the Nrf2-(Keap1) pathway [235].

### 8.9. Luteolin

Luteolin is a natural flavonoid abundantly present in many plant species. It is mainly found in vegetables and fruits, such as sweet bell peppers, carrots, onion leaves, broccoli, parsley, chrysanthemum flowers, and celery [236,237]. It was isolated from *Platycodon grandiflorum, Perilla frutescens, Apium graveolens, Cajanus cajan, Apium graveolens, Mentha spicata, and Sesbania grandifolra* [238]. Luteolin was utilized in the treatment of prostate, breast, pancreatic, skin, colon, oral, lung, ovarian, and kidney cancers [239]. It has a potent cytotoxic effect against different breast cancer cell lines, including MDA-MB-231 and BT5-49 [239]. It obstructed cell proliferation and stimulated apoptosis of H460 and A549 cells [240,241,242]. It inhibited metastasis, angiogenesis, and cell proliferation, and stimulated apoptosis through various mechanisms [243]. The extrinsic and intrinsic apoptosis pathways were stimulated and the death receptor 5 expression was improved by luteolin [244]. Stimulating JNK and obstructing translocation of NF-κB were mediated the cellular growth inhibition, G2 arrest, and apoptotic cell death induction [245]. Furthermore, luteolin inhibited cancer cell proliferation by angiogenesis inhibition via blocking VEGF receptor stimulation and its downstream molecule PI3K/Akt and PI3K/p70S6 kinase pathways [246].

Colon cancer and diabetes are the primary cause of death worldwide [247]. Various luteolin signaling pathways, including SOD, p53, eNOS, Wnt, iNOS, MMP9, and the cyclin-CDK pathway, are used to treat cancer/diabetes [247]. Since chronic hyperglycemia produces an intracellular level of reactive oxygen species, luteolin has anti-diabetic activity due to its antioxidant properties [247]. Moreover, luteolin can regulate diabetes through mammalian targets of rapamycin (mTOR), cytokine, AMPK, and p53 [247].

### 8.10. Quercetin

Quercetin is a polyphenolic flavonoid found in apples, berries, broccoli, onions, broccoli, green tea, red tea [248], *Aesculus hippocastanum, Ginkgo biloba, and Hypericum perforatum* [249]. The biological activities of quercetin are anticancer, antidiabetic, antiobesity, neuroprotective, antimicrobial, antiviral, hepatoprotective, and anti-inflammatory activity [250]. Quercetin stimulated apoptosis and inhibited cellular growth through the reduction of epidermal growth factor receptor (EGFR) expression [251]. It induced apoptosis by directly upregulating caspase-3 and-9 and proapoptotic Bcl-2 family members, but it also induced apoptosis by directly downregulating antiapoptotic Bcl-xL [252]. Moreover, it possessed a cytotoxic effect against different breast cancer cell lines, including MDA-MB-231 and MCF-7 via apoptosis activation along with G1 phase arrest [253,254]. Quercetin improved the chemosensitivity of breast cancer cells to doxorubicin by obstructing proliferation and invasion of cells, leading to apoptosis activation [255]. It also has good cytotoxic effects against human lung carcinoma A549 cells as it can inhibit cell invasion and migration, stimulate apoptosis, and decrease the number of tumor cells [256]. A study showed that 16 weeks of administration of quercetin at a concentration of 200 and 400 mg/kg can decrease the number and the tumor size of papillomas in skin tumors prompted by croton oil in Swiss albino mice [257].

Quercetin can decrease serum glucose level through several mechanisms, including antioxidant action, modulating hepatic gene expressions, obstructing α-glucosidase activity in vitro, and improving insulin action along with skeletal muscle mitochondrial biogenesis enhancement [258]. It was utilized to reduce blood glucose and urine sugar levels, and increase plasma insulin and hemoglobin levels [259]. Quercetin was found to be an effective PPAR-γ inhibitor [259].

### 8.11. Berberine

Berberine isolated from *Coptis japonica* Makino, *Coptis chinensis* Franch, and *Berberis aristata* Sims is an essential isoquinoline alkaloid. It is found in barberry, goldenseal, tree turmeric, and oregon grape [260]. It is a pharmacologically significant secondary metabolite as it can help in the treatment of cancer, diabetes, and obesity [260].

Colorectal, lung, liver, ovarian, prostate and cervical cancers are all inhibited by berberine [260]. Under illumination with a black light lamp (320,450 nm; 20 W), SGC803 gastric cancer cells combined with berberine (10 g/mL) showed a good inhibitory proliferation effect. Additionally, berberine combined with 5-ALA-photodynamic therapy inhibits MGC-803 gastric cancer cell proliferation, reduces survivin and Bcl2 expression, stimulates cell apoptosis, and upregulates p53 and Bax expression. These findings imply that berberine has the ability to be utilized in conjunction with photosensitizers [260]. Activating apoptosis and autophagy, inhibiting cell proliferation, and altering protein expression are berberine-photodynamic therapy’s mechanisms in treating tumors [260]. Furthermore, berberine was utilized against breast cancer as it improved JNK phosphorylation, activated caspase-3, reduced the mitochondrial membrane potential, decreased the expression of Bcl-2, and increased the release of cytochrome c and AIF [261].

Berberine decreased blood glucose and the risk of metabolic syndrome, improved insulin sensitivity, stimulated weight loss, improved lipid metabolism, reduced the levels of hemoglobin A1C and triglyceride, increased the mRNA expression of adiponectin, and reduced leptin and resistin secretion [262]. Reduction of body weight, body mass index, and waist circumference was revealed after the administration of 500 mg of berberine either two or three times a day for three months [262].

### 8.12. Phytosterols

Phytosterols are plant-derived lipid compounds that are similar to cholesterol but differ in their carbon side chains and the presence or absence of a double bond. They are divided into two categories which are sterols (unsaturated compounds) and stanols (saturated molecules) [263]. Phytosterols are commonly found in plants or macro fungi and have a number of pharmacological activities, including anticancer, hypolipidaemic, anti-inflammatory, and antidiabetic activity [263].

Phytosterols can be utilized to improve breast, ovary, liver, lung, prostate and stomach cancers. They have several suggested mechanisms, including inhibition of carcinogen production, angiogenesis, cancer cell growth, multiplication, invasion, and metastasis and induction of apoptosis and cell cycle arrest [264]. Female SCID mice were fed specified diets supplemented with 2% phytosterols, 2% cholesterol, or a 0.2% cholic acid vehicle to evaluate the effects of phytosterols on breast cancer cell growth and metastasis [264]. After 2 weeks, mice were injected with MDA-MB-231 cells into their inguinal mammary fat pads. Eight weeks after the dietary supplementation, mice given phytosterols had a 40% reduction in serum cholesterol levels [264]. Tumor sizes were 33% lower in animals fed the phytosterol diet compared to those fed the cholesterol diet [264]. Tumor cell metastasis to lymph nodes and lungs was 57% of the phytosterol-fed animals and 71% of the cholesterol-fed animals [264]. Another study showed that β-sitosterol enriched diet decreased tumor size in ovariectomized athymic mice injected with MCF-7 cells by 32–42% [264].

Phytosterols have been shown to substantially lower total cholesterol (TC) and low-density lipoprotein cholesterol (LDL-C) in the blood, and rise the HDL-C/LDL-C and HDL-C/TC indexes [265]. Ergosterol could be a promising hypoglycemic agent for the treatment of type 2 diabetes, with a mechanism of stimulating GLUT4 translocation and expression through the PI3K/Akt and PKC pathways [265]. Ergosterol was utilized to improve insulin resistance and blood lipid indices, and decrease fasting blood glucose levels. Furthermore, the phosphorylation of Akt and PKC was improved in different tissues [265]. Table 2 summarizes the anticancer and antidiabetic mechanisms for selected natural products that target both diseases.

## 9. The Controversy of Exogenous Antioxidants Administration in Cancer

Research and studies about the role of reactive oxygen species (ROS) and the effect of exogenous administration of antioxidants’ supplements in cancer are opposed with controversy and conflicting outcomes. On one hand, antioxidants are commonly used by cancer patients and healthy individuals as a cancer-fighting strategy [269,270,271]. Clinical trials with antioxidants, on the other hand, have shown contradictory findings; some trials showed that antioxidants raised cancer risks [272,273,274,275].

Klein A et al. conducted a study between 2004 and 2011 and found that prostate cancer risk for healthy men was markedly increased upon supplementation with 400 IU/day of vitamin E supplement [276], whereas VI Sayin et al. clarified that supplementation with the antioxidants *N*-acetylcysteine (NAC) and vitamin E noticeably enhances human lung cancer cells proliferation and tumor progression in mice with B-RAF- and K-RAS-induced lung cancer via decreasing ROS, DNA damage, and p53 levels. Additionally, Volkan S. et al. showed that the antioxidants supplementation may accelerate early tumors or precancerous lesions growth in elevated-risk populations (like smokers and chronic obstructive pulmonary disease patients who receive NAC to reduce mucus production) because the somatic p53 mutations occur late in the tumor progression process [277]. Another study by Clotilde W. et al. demonstrated that long-term supplementation of NAC and vitamin E stimulates K-RAS-induced lung cancer metastasis by decreasing free heme levels and stabilizing the pro-metastatic basic leucine zipper transcription factor 1 (BACH1). BACH1 stimulates glycolysis-dependent metastasis of mouse and human lung cancer cells by activating transcription of Hexokinase 2 and Gapdh and increasing glucose uptake, glycolysis rates, and lactate secretion [278]. Moreover, Le Gal et al. studied the effect of antioxidants on malignant melanoma in a mouse model and showed that the administration of NAC and Trolox (the soluble vitamin E analog) expand lymph node metastases and cause increased glutathione-synthesis-dependent invasiveness [279]. Indicating that active tumors preserve ROS levels within limits that stimulate proliferation without leading to cytotoxicity [280]. Similar results of melanoma metastasis were found in a different mouse model (NOD-SCID *Il2rg^−/−^* (NSG) mice) when supplemented regularly with subcutaneous injection of NAC [281]. Clinical trials involving the use of antioxidants combined with chemotherapy are summarized in Table 3.

In conclusion, the supplementation of exogenous antioxidants may aid cancer cells to navigate the metastatic cascade and adopt adaptive steps to counteract the beneficial effect of antioxidant supplements. Nevertheless; additional clinical studies are required to confirm the generalizability of these remarks and to expand them to practical application especially with existing cancer patients.

## 10. Conclusions

Cancer and diabetes represent global health challenges and the number of patients is contentiously increasing for both diseases. Hyperglycemia, hyperinsulinemia, and imbalanced fat metabolism in diabetic patients increase the altered metabolism in cancer cells. This metabolic link between cancer and diabetes complicates therapeutic protocols and reduces survival rates. Natural products with their diverse mechanisms of action can be used as adjuvant therapy to enhance conventional treatments. These natural products act by targeting interconnected mechanisms between cancer and diabetes. Further research is needed to explore the potential use of natural products in combination therapies to treat cancer in diabetic patients.

## Figures and Tables

**Table 1:** Various definitions of metabolic syndrome.

**Risk Factors**	**NCEP/ATP III**	**WHO 1999**	**IDF**
	(3 of 5 criteria necessary) [41,42]	(impaired glucose regulation or hyperinsulinemia and 2 or more criteria necessary) ^A^	(increased waist circumference plus any 2 of other 4 criteria) [50]
**Fasting glucose**	≥110 mg/dL	≥110 mg/dL	≥110 mg/dL
**Prandial glucose**		>140 mg/dL	
**Hyperinsulinemia**		Fasting serum insulin: third quartile for the control group	
**Hypertriglyceridemia ^B^**	≥150 mg/dL	≥150 mg/dL	≥150 mg/dL
**Low HDL-C**	<40 mg/dL <50 mg/dL	<35 mg/dL <39 mg/dL	<40 mg/dL <50 mg/dL
**Men** **Women**			
**Abdominal obesity**	>102 cm >88 cm	waist: hip ratio >0.9 in >0.85 in	≥94 cm ≥80 cm
**Men** **Women**			
**Hypertension ^B^**	≥130/≥85 mm Hg	≥140/≥90 mm Hg	≥130/≥85 mm
**Micro-albuminuria**		≥20 μg/min	

^A^ World Health Organization. Definition, diagnosis, and classification of diabetes mellitus and its complications: report of a WHO consultation. Part 1: diagnosis and classification of diabetes mellitus. Geneva, Switzerland: World Health Organization, 1999. ^B^ Considered positive criteria if a person is on medications for lipids, hypertension, or hyperglycemia regardless of value. Abbreviations: NCEP/ATP III, National Cholesterol Education Program/Adult Treatment Panel III; WHO, World Health Organization; IDF, International Diabetes Federation; HDL, high-density lipoprotein.

**Table 2:** Antidiabetic and anticancer activity of some natural products that have the ability to treat both diseases.

Natural Products	Mechanism of Action in Cancer	Mechanism of Action in Diabetes
**Resveratrol**	↑ caspase-3 and caspase-9, p53, X-associated protein Bcl-2 in human cervical carcinoma [141] ↓ signaling pathway of AKT/STAT3 in colon cancer cells [142] ↑ TRPM2 channel in glioblastoma cells [143] control the expressions of vimentin, CTA-2, IL-1β, TNF- α, and N-cadherin in pancreatic cancer cells [144] ↑ Bcl-2 activity, p53, Bax, and caspase expression in breast cancer [145] ↓ tumor volume in Ehrlich ascites carcinoma cells [148]	↓ α-glucosidase, carbohydrate absorption, and post-prandial blood glucose response in mice [149] ↑ muscle and liver glucose absorption ↓ adipose tissue and liver inflammation ↑ adaptive thermogenesis capability ↓ pancreatic insulin secretion, and mimic caloric restriction effects [149] ↑ serum blood glucose, insulin level, and dyslipidemia in diabetic rats [151]
**Curcumin**	↑ apoptotic ligand-inducing tumor-necrosis-factor-related apoptosis (TRAIL) pathways ↑ death receptor factor 5in HT-29 and HCT-116 colon cancer cells [161] ↑ Fas-mediated apoptotic pathway, caspase 8 activation, Bax expression ↓ Bcl-2 in HT-29 colon cancer [162,163], and HCT-116 [155] ↓ JAK/STAT signal in osteosarcoma [164] interfere with nuclear factor κ (NFκB), epidermal growth factor receptor (EGFR), and mitogen-activated protein kinase (MAPK) in prostate cancer [165]	↑ adipocyte differentiation substantially, glucose levels, and the activity of human peroxisome proliferator-activated receptor (PPAR)-gamma ligand-binding [166] ↓ glucose uptake, leptin levels, NF-kB p65 nuclear fraction, phospho-JNK1/2, phospho-IRS-1(S), MMP-2, MMP-9 and VEGF protein in 3T3-L1 adipocytes [166] ↓ levels of urinary enzymes (acid phosphatase, alkaline phosphatase (ALP), aspartate aminotransferase (AST), and alanine aminotransferase (ALT)) [167] ↓ JAK/STAT signalling pathway ↓ myocardial degeneration, lipid peroxidation levels, IL-6, creatine kinase-MB (CK-MB), troponin I, and tumor growth factor-β1 (TGF-β1) in co-treatment with metformin [168]
**Thymoquinone**	↓ cell arrest at various stages in MCF-7 cell line [175] ↓ serum TNF- α, IL-6, and iNOS enzyme production and improve histopathological outputs in Wistar rats [176] ↓ phosphorylation of EGFR to tyrosine-1173 residues and JAK2 in HCT 116 [177] ↑ cytotoxicity in the xenograft mouse model of breast cancer [178] ↑ apoptosis by reducing antiapoptotic protein expression [179]	↓ blood glucose level, triglycerides and cholesterol concentrations ↑ high-density lipoprotein, insulin sensitivity and pancreatic β-cell regeneration [180] maintain the integrity of pancreatic β-cells by enhancing oxidative stress [181]
**EGCG**	↓ MMP-2 through modulation the Src signaling pathway in cervical cancer [190] ↓ cyclinD1 ↑ LIMD1, RBSP3, and p16 cell-cycle inhibitors at the G1/S cell cycle level in cervical cancer cell line [192] ↑ AKT/STAT3 pathway, apoptosis in cisplatin-resistant oral cancer cell [193]	↓ α-glucosidase ↑ glucose uptake ↑ GLUT4 translocation to plasma membrane through PI3K/AKT signaling pathway in L6 skeletal muscle cells [195]
**Allicin**	↓ cell migration, STAT3 ↑ SHP-1, apoptosis in cholangiocarcinoma cell [198] ↓ signaling pathway of NF-κ in colorectal cancer [199] ↓ cytokine release ↑ p53 and radiotherapy sensitivity enhancement in glioma cells [200] ↑ apoptosis ↓ ornithine decarboxylase in neuroblastoma Cells. [201] ↓ MMP-9 mRNA expression ↑ cyclin D1 in melanoma cells [202]	↑ insulin levels ↓ hyperglycemia, (GLUT4) and IRSs expression [204] ↑ Nrf2 ↓ Keap1, HIF-1α, SBP, and VEGF [204] ↓ autoantibodies and anti-islet cell antibodies (ICA) for type 1 diabetes (IDDM) [205]
**Emodin**	↑ apoptosis, cell cycle arrest, HIF-1α, glutathione phase I and II, and glutathione S-transferase P, N-acetyltransferase ↓ angiogenesis, invasion, migration, formation of chemical-induced carcinogen-DNA adducts, HER2/neu, CKII kinase, and p34cdc2 kinase in human cancer cells [215] ↓ ERK phosphorylation in epithelial ovarian cancer [266]	↓ cellular FLICE-inhibitory protein (cFLIP) and p38MAPK pathway ↑ activate PPARγ pathway, and modulate AMPK signalling pathway [216]
**Genistein**	↓ proliferation, angiogenesis and metastasis ↑ apoptosis leading to tumor growth reduction in hepatocellular and gastric cancer models of Wistar rats [219,220] ↓ COX-2-stimulating factors such as activated protein-1 (AP-1) and Nf-κB in pancreatic, colon, breast, and lung cancer [217]	modulate the proliferation of β-cells and the secretion of insulin [222] ↑ cAMP signaling, and the mass of islets in diabetic mice [223]
**Parthenolide**	interrupt DNA replication [267] ↓ STAT3 ↑ apoptotic pathway [268] ↑ p53 and reactive oxygen species (ROS) [228] ↓ breast cancer stem-like cells by stimulating oxidative stress and necrosis [231] ↓ cell growth of glioblastoma cells [232]	↓ inflammation and remodel the impaired insulin signaling pathway ↑ cubilin and albumin uptake expression [234] modulate reactive oxygen species production, and control the Nrf2- (Keap1) pathway [235] ↓ adipogenic factors (PPARc and C/EBPa) and its target protein FABP4 production [235]
**Luteolin**	↑ apoptosis pathways and death receptor 5 expression [244] ↑ JNK ↓ NF-κB, mediate the cellular growth inhibition and G2 arrest [245] ↓ proliferation, angiogenesis, VEGF receptor, PI3K/Akt and PI3K/p70S6 kinase pathways [246] ↓ cell proliferation and stimulate apoptosis in H460 and A549 cells [240,241,242]	regulate diabetes through mammalian target of rapamycin (mTOR), cytokine, AMPK, and p53 [247]
**Quercetin**	↑ apoptosis ↓epidermal growth factor receptor (EGFR) expression [251] ↑ caspase-3 and-9 ↓ Bcl-2, Bcl-xL [252] ↑ G1 phase arrest in breast cancer cell lines [253,254] ↓ cell invasion and migration ↑ apoptosis in human lung carcinoma A549 cells [256]	↓ serum glucose level modulate hepatic gene expressions ↓ α-glucosidase activity, and PPAR-γ ↑ insulin action [259]
**Berberine**	↓ survivin and Bcl2 ↑ apoptosis, p53, and Bax expression in gastric cancer cells [260] ↑ JNK phosphorylation, cytochrome c and AIF, and caspase-3 ↓ mitochondrial membrane potential, and Bcl-2 expression in breast cancer [261]	↓ blood glucose ↓ risk of metabolic syndrome, levels of hemoglobin A1C and triglyceride ↓ leptin and resistin secretion ↑ insulin sensitivity and weight loss ↑ lipid metabolism and mRNA expression of adiponectin [262]
**Phytosterols**	↓ cell growth and metastasis ↓ tumor size in athymic mice injected with MCF-7 cells [264]	↑ GLUT4 translocation ↑ PI3K/Akt and PKC pathways ↓ fasting blood glucose levels [265]

**Table 3:** Clinical trials conducted using combination treatment of antioxidants with chemotherapeutic agents.

Combination Treatment	Results	Ref.
Vitamin C + doxorubicin	Increased resistance to treatment in cell lines of chronic myelogenous leukemia (K562) and lymphoma (RL)	[282]
	Larger tumors in mice with RL cell xenografts	
Resveratrol + paclitaxel	Decreased antitumor action of paclitaxel in human breast tumor cells	[283]
Quercetin at low doses + cisplatin, 5-FU, taxol or pirarubicin	Decreased efficiency of the treatment in athymic nude mice with ovarian tumor cells (C13*) xenografts	[284]
*N*-acetylcysteine before or up to 1 h after the drug + cisplatin	Blockade of proapoptotic effect of cisplatin in human ovarian carcinoma cells (SKOV3), human SCLC tumor cells (B.5 LX-1), human glioblastoma cells (U87), and rat fibroblasts	[285]
